# Machine Learning for 1-Year Mortality Prediction in Lung Transplant Recipients: ISHLT Registry

**DOI:** 10.3389/ti.2025.14121

**Published:** 2025-06-26

**Authors:** Hye Ju Yeo, Dasom Noh, Eunjeong Son, Sunyoung Kwon, Woo Hyun Cho

**Affiliations:** ^1^ Transplant Research Center, Research Institute for Convergence of Biomedical Science and Technology, Pusan National University Yangsan Hospital, Yangsan, Republic of Korea; ^2^ Department of Internal Medicine, School of Medicine, Pusan National University, Busan, Republic of Korea; ^3^ Department of Information Convergence Engineering, Pusan National University, Yangsan, Republic of Korea; ^4^ School of Biomedical Convergence Engineering, Pusan National University, Yangsan, Republic of Korea; ^5^ Center for Artificial Intelligence Research, Pusan National University, Busan, Republic of Korea

**Keywords:** lung transplantation, mortality, machine learning, risk factors, prediction model

## Abstract

Optimizing lung transplant candidate selection is crucial for maximizing resource efficiency and improving patient outcomes. Using data from the International Society for Heart and Lung Transplantation (ISHLT) registry (29,364 patients), we developed a deep learning model to predict 1-year survival after lung transplantation. Initially, 25 pretransplant factors were identified, and their importance was assessed using SHapley Additive exPlanations values. We refined the model by selecting the top 10 most influential factors and compared its performance with the original model. Additionally, we conducted external validation using an independent in-house dataset. Among the 29,364 patients, 4,729 (16.1%) died within 1 year, while 24,635 survived. The Gradient Boosting Machine (GBM) model achieved the highest performance (AUC: 0.958, accuracy: 0.949). Notably, the streamlined model using only the top 10 factors maintained identical performance (AUC: 0.958, accuracy: 0.949). The in-house dataset used for external validation showed significant compositional differences compared to the ISHLT dataset. Despite these differences, the GBM model performed well (AUC: 0.852, accuracy: 0.764). Notably, the Multilayer Perceptron model demonstrated superior generalization with an AUC of 0.911 and accuracy of 0.870. Our machine learning-based approach effectively predicts 1-year mortality in lung transplant recipients using a minimal set of pretransplant factors.

## Graphical Abstract

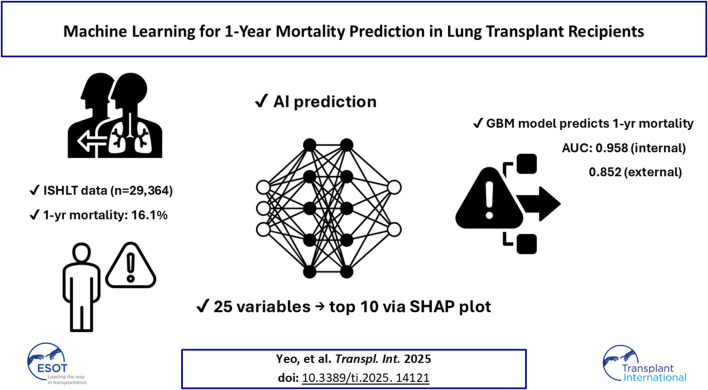

## Introduction

Lung transplantation is a critical intervention for patients with end-stage lung disease, providing significant survival benefits. As the population ages and medical technologies advance, the demand for lung transplants continues to rise [1]. However, the global supply of organ donors is insufficient to meet this growing demand [2]. Despite advancements in surgical techniques and immunosuppressive therapies, the post-transplant environment remains challenging, with various factors influencing outcomes [3]. Given the scarcity of medical resources, prioritizing patients with a low risk of mortality post-transplant is imperative. Predicting 1-year mortality following lung transplantation is, therefore, a critical goal to optimize patient care and enhance clinical decision-making.

Traditional methods for predicting mortality after lung transplantation have primarily relied on clinical judgment and risk-scoring systems [4–7]. While these scoring systems aid in prioritizing candidates, they often fail to account for the complexity of individual patient trajectories. This is due to the limited number of variables they include and their heavy reliance on clinical judgment. Additionally, several factors traditionally considered important, such as recipient age, body mass index (BMI), and the duration of preoperative mechanical ventilation, have not consistently shown the expected prognostic impact in previous studies [8]. A recent meta-analysis found that only one factor, postoperative extracorporeal membrane oxygenation (ECMO) use, was significantly associated with 1-year mortality, while other commonly accepted risk factors demonstrated minimal prognostic significance [8]. These limitations emphasize the need for more accurate, personalized risk prediction methods, as existing models may lack the granularity required for individualized treatment.

In this context, machine learning approaches offer the potential to develop predictive algorithms that can integrate diverse patient data and identify subtle patterns that traditional methods may overlook. Machine learning models can enhance pretransplant risk stratification, assist clinicians in selecting and counseling candidates, guide post-transplant surveillance strategies, and inform interventions to mitigate adverse outcomes. By providing timely insights into individual patient trajectories, these models can improve resource allocation and patient-centered care. Despite their potential, however, machine learning models predicting patient prognosis post-lung transplantation have not been extensively studied [9–11]. In this study, we developed and validated a machine learning-based model to predict 1-year mortality among lung transplant recipients using data from the International Society for Heart and Lung Transplantation (ISHLT). Additionally, we performed external validation using the in-house dataset from our hospital.

## Materials and Methods

**FIGURE 1:**
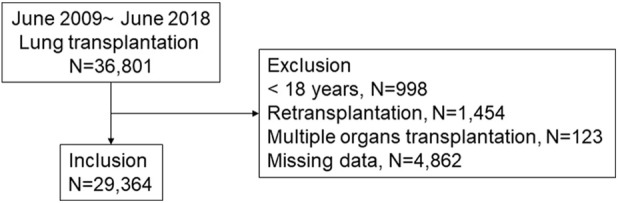
Patient inclusion. A total of 29,364 lung transplant patients were included in this study.

**FIGURE 2:**
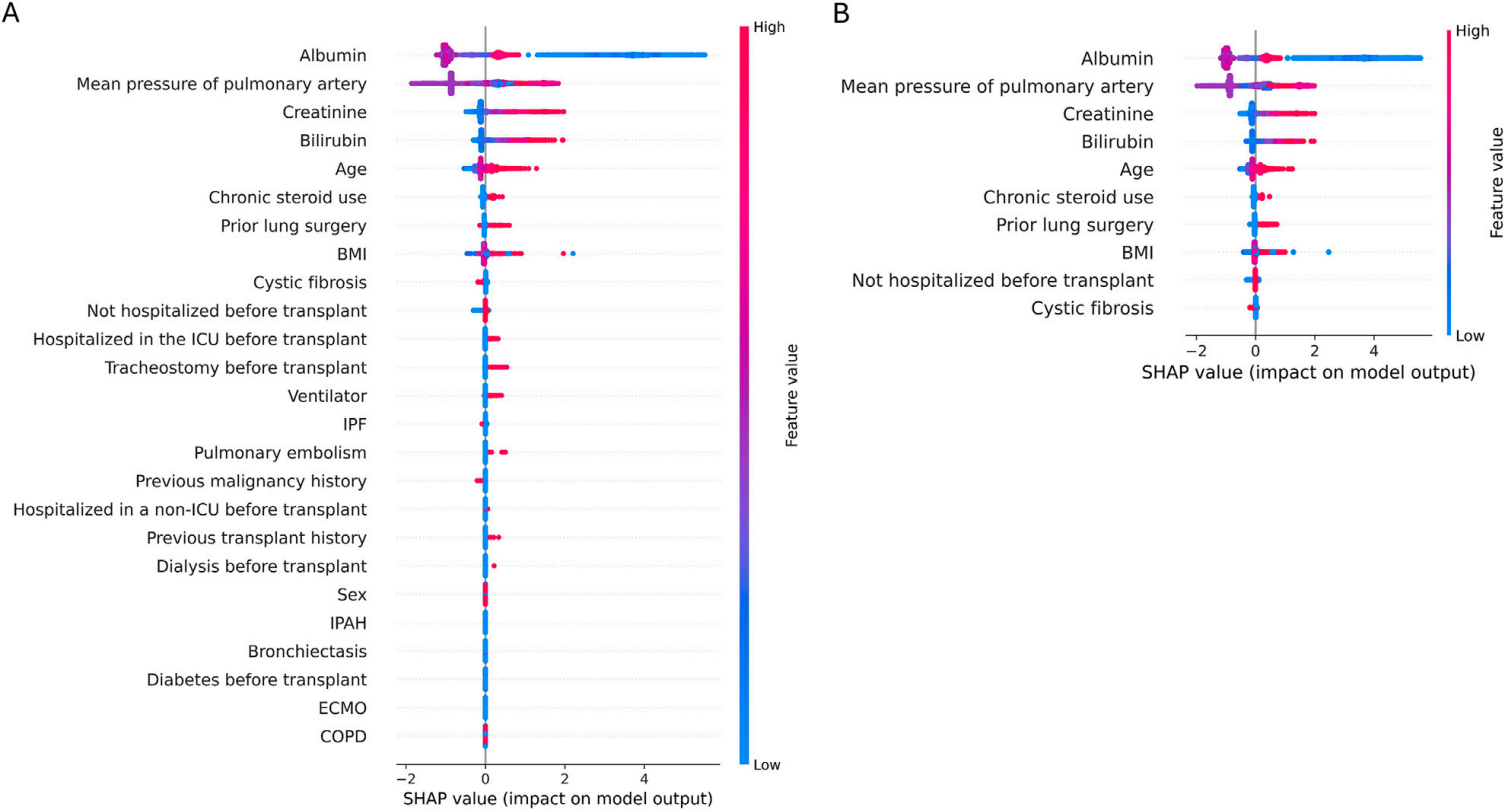
Shapley Additive explanations (SHAP). A SHAP plot illustrates the influence of each feature’s value on the prediction outcome, with red indicating an increase in prediction and blue indicating a decrease. It demonstrates notable impacts in the following sequence: albumin, mean pulmonary artery pressure, and functional status. Increasing albumin levels correlate with decreased mortality (blue), while higher mean pulmonary artery pressure correlates with increased mortality (red). **(A)** SHAP plot of 25 features, **(B)** SHAP plot of 10 features.

Patients who underwent lung transplantation and who were registered in the ISHLT registry from June 2009 to June 2018 were included (Figure 1). By early 2019, 45 centers worldwide had submitted data to the ISHLT International Thoracic Organ Transplant Registry using a secure, web-based data entry system. Detailed spreadsheets of the data elements collected in the Registry are available on the ISHLT International thoracic organ transplant website^1^. We excluded patients < 18 years, those who had undergone retransplantation or multiple organ transplants, and those with unavailable follow-up status or missing data. Consequently, 29,364 patients were available for analysis. The ethics committees and review board of Pusan National University Yangsan Hospital (PNUYH) (55-2024-128) approved the current study, and informed consent was waived due to the retrospective nature of the study. This study adhered to the Transparent Reporting of a Multivariable Prediction Model for Individual Prognosis or Diagnosis (TRIPOD) reporting guideline. Initially, key variables were selected through regression analysis to develop a predictive model for 1-year mortality following lung transplantation. This analysis identified 25 variables significantly associated with 1-year mortality. We then evaluated feature importance using SHapley Additive exPlanations (SHAP) values (Figure 2A) (Supplementary Figure S1) and developed a refined model incorporating the top 10 most important features. The performance of this refined model was compared with that of the original model, which included all 25 variables. Finally, external validation was conducted using our hospital’s in-house dataset.

### Machine Learning-Based Model Development

We employed six machine learning models to predict 1-year mortality after lung transplantation: Logistic Regression (LR) [12], Support Vector Machine (SVM) [13], Random Forest (RF) [14], Gradient Boosting Machine (GBM) [15], Balanced Random Forest (BRF) [16], and a neural network model, Multilayer Perceptron (MLP) [17]. To evaluate model performance, we conducted five-fold cross-validation. The entire dataset was divided into five subsets, and during each iteration, one subset was used for validation while the remaining four were used for training. This process was repeated until each subset had been used for validation once, and the final results were reported as the average performance across all five iterations (Supplementary Figure S2). To interpret the predictions of our models, we employed SHAP [18], a widely used method for estimating the contribution of each feature to a prediction. SHAP provides explanations based on Shapley values derived from game theory. Specifically, we utilized the Python SHAP package [18] and applied the TreeExplainer for GBM. We used the scikit-learn package [19] for most models, except for BRF, which was implemented using the imbalanced-learn package [20]. Default model settings were applied without additional hyperparameter tuning. However, due to class imbalance in the ISHLT dataset, class weight adjustments were applied for LR, RF, SVM, and BRF to account for the imbalance. Additionally, survival analysis based on model predictions was performed using the lifelines Python package [21]. Finally, external validation of the developed models was conducted using our hospital’s in-house dataset, further assessing their generalizability and robustness.

### Statistical Analyses

Variables with excessive missing values (defined as more than 20% missing data) were removed, and patient samples with any missing data were excluded. This approach ensures that the analysis is based on complete cases, minimizing potential biases from missing data. Continuous variables are reported as either the mean ± standard deviation or the median with interquartile range (IQR), depending on their distribution. Statistical comparisons of continuous variables were performed using the Student’s t-test or the Mann-Whitney U test, as appropriate. Categorical variables are expressed as frequencies and percentages and analyzed using the chi-square or Fisher’s exact test, as appropriate. To identify factors associated with 1-year mortality after lung transplantation, we conducted univariate regression analysis. Additionally, we validated the GBM model by comparing the actual survival curves of patients, grouped based on the model’s predictions. Patients predicted by the model to die were classified into the high-risk group (death group), and those predicted to survive were classified into the low-risk group (survival group). Survival curves for these two groups were illustrated using Kaplan-Meier plots, and the log-rank test was performed to compare survival rates between the groups. All statistical analyses were performed using R software (version 4.2.0; R Foundation for Statistical Computing, Vienna, Austria^2^). Statistical significance was set at P < 0.05.

## Results

### Patient Characteristics

Among the 29,364 patients included in the study, 4,729 (16.1%) died within 1 year, whereas 24,635 survived. The baseline pretransplant characteristics of the patients are summarized in Table 1. Their average age was 53.4 ± 13.4 years, with 17,032 (58%) males, and the average body mass index (BMI) was 24.9 ± 4.5. The primary diagnoses were as follows: chronic obstructive pulmonary disease (COPD) in 27.9%, idiopathic pulmonary fibrosis (IPF) in 26.8%, cystic fibrosis in 14.6%, nonidiopathic interstitial pneumonia (nonIIP) ILD in 7.3%, nonIPF idiopathic interstitial pneumonia (IIP) in 4.7%, alpha-1 antitrypsin deficiency in 3.4%, idiopathic pulmonary arterial hypertension (IPAH) in 2.8%, noncystic fibrosis (nonCF) bronchiectasis in 2.6%, sarcoidosis in 2.5%, nonIPAH pulmonary hypertension in 1.7%, connective tissue disease-associated ILD (CTDILD) in 1.3%, obliterative bronchiolitis in 0.9%, lymphangioleiomyomatosis in 0.8%, and other diagnoses in 2.6%. Pretransplant, 1,757 patients (6%) were admitted to the intensive care unit (ICU), 1,515 (5.2%) were hospitalized in general wards, and 26,092 (88.9%) were not hospitalized. Additionally, 1,021 patients (3.5%) were on ventilators, and 642 (2.2%) were on extracorporeal membrane oxygenation (ECMO). Pretransplant diabetes was present in 3,288 patients (11.2%), pretransplant dialysis was performed in 61 patients (0.2%), and 64 patients had a history of organ transplantation other than the lung. Additionally, 781 (2.7%) and 2,601 (8.9%) patients had previous heart and lung surgeries, respectively.

**TABLE 1:** Baseline characteristics of patients before the transplant.

Variable	Survivors (N = 24,635)	Death (N = 4,729)	P
Age, years	53.2 ± 13.5	54.5 ± 13.1	<0.001
Male	14,186 (57.6)	2,846 (60.2)	0.001
BMI, kg/m^2^	25.3 ± 4.3	23.1 ± 4.9	<0.001
Total bilirubin, mg/dL	0.6 ± 0.9	2.0 ± 2.0	<0.001
Creatinine, mg/dL	0.8 ± 0.3	1.7 ± 1.0	<0.001
Albumin, g/dL	3.7 ± 0.4	3.1 ± 0.6	<0.001
Diagnosis			
COPD	7036 (28.6)	1,171 (24.8)	<0.001
Alpha-1 antitrypsin deficiency	847 (3.4)	151 (3.2)	0.394
Cystic fibrosis	3,782 (15.4)	516 (10.9)	<0.001
Non-cystic fibrosis bronchiectasis	616 (2.5)	147 (3.1)	0.016
IPF	6,425 (26.1)	1,438 (30.4)	<0.001
IIP, non-IPF	136 (4.6)	254 (5.4)	0.024
ILD, non-IIP	1803 (7.3)	352 (7.4)	0.763
CTD ILD	319 (1.3)	52 (1.1)	0.271
Sarcoidosis	612 (2.5)	134 (2.8)	0.162
Lymphangioleiomyomatosis	200 (0.8)	35 (0.7)	0.612
Idiopathic pulmonary artery hypertension	652 (2.6)	184 (3.9)	<0.001
Pulmonary hypertension- not idiopathic	385 (1.6)	102 (2.2)	0.003
Obliterative bronchiolitis	236 (1.0)	29 (0.6)	0.022
Other	586 (2.4)	164 (3.5)	<0.001
Diabetes	2,826 (11.5)	462 (9.8)	0.001
Malignancy history	1,205 (4.9)	196 (4.1)	0.027
Ventilator use	818 (3.3)	203 (4.3)	0.001
ECMO use	508 (2.1)	134 (2.8)	0.001
Prior cardiac surgery	637 (2.6)	144 (3.0)	0.072
Dialysis	45 (0.2)	16 (0.3)	0.031
Prior lung surgery	2,234 (9.1)	367 (7.8)	0.004
Chronic steroid use	6,185 (25.1)	1,079 (22.8)	0.001
Tracheostomy before transplant	484 (2)	122 (2.6)	0.006
Previous transplant history except lung	48 (0.2)	16 (0.3)	0.053
Pulmonary embolism	60 (0.2)	24 (0.5)	0.002
FEV1	39.2 ± 20.9	40.7 ± 20.1	0.002
Mean pulmonary artery pressure, mmHg	27.9 ± 8.4	32.4 ± 9.1	<0.001
Medical condition before transplant			<0.001
ICU admission	1,401 (5.7)	356 (7.5)	
General ward admission	1,304 (5.3)	211 (4.5)	
No admission	21,930 (89)	4,162 (88)	

Data are presented as means ± SD, or number (%).

### Machine Learning-Based Model Performance and Model Interpretation

We performed univariate regression analysis of 1-year mortality. Table 2 presents the odds ratio of the 25 factors used in the model. We evaluated the performance of six machine learning models with 25 pretransplant features for predicting 1-year mortality after lung transplantation. The results are presented in Table 3. We used area under the curve (AUC), accuracy, sensitivity, specificity, positive predictive value, and negative predictive value as evaluation metrics. Most models exhibited high performance, with AUC and accuracy either surpassing or closely approaching 0.9. The GBM model achieved the highest performance, with an AUC and accuracy of 0.958 and 0.949, respectively.

**TABLE 2:** Univariate associations of pretransplant characteristics with 1-year survival.

Variable	OR (95% CI)	P
Age	1.01 (1.00–1.01)	<0.001
Male	1.10 (1.04–1.17)	0.001
Prior lung surgery	1.17 (1.06–1.31)	0.003
any previous transplantation history	1.67 (1.03–2.74)	0.040
Albumin	0.22 (0.21–0.22)	<0.001
Chronic steroid use	0.88 (0.83–0.95)	<0.001
Diabetes	1.20 (1.09–1.32)	<0.001
total bilirubin	1.09 (1.09–1.10)	<0.001
BMI	0.90 (0.90–0.91)	<0.001
creatinine	1.32 (1.31–1.33)	<0.001
Previous malignancy history	1.18 (1.03–1.37)	0.021
mean pulmonary artery pressure	1.04 (1.04–1.04)	<0.001
non-hospitalized before transplant	0.91 (0.82–1.00)	0.043
non-ICU hospitalized before transplantation	0.84 (0.72–0.97)	0.018
ICU hospitalized before transplantation	1.35 (1.20–1.52)	<0.001
Ventilator	1.27 (1.10–1.46)	0.001
ECMO	1.36 (1.14–1.61)	<0.001
COPD	0.82 (0.77–0.88)	<0.001
Cystic fibrosis	0.68 (0.61–0.75)	<0.001
IPF	1.49 (1.26–1.76)	<0.001
non-CF bronchiectasis	1.25 (1.04–1.50)	0.016
IPAH	1.43 (1.23–1.66)	<0.001
Dialysis	1.80 (1.10–2.94)	0.019
Pulmonary embolism before transplant	1.93 (1.29–2.88)	0.001
Tracheostomy before transplantation	1.29 (1.08–1.54)	0.006

OR; odds ratio, CI; confidence interval, BMI; body mass index, ICU; intensive care unit, ECMO; extracorporeal membrane oxygenation, COPD; chronic obstructive pulmonary disease, IPF; idiopathic pulmonary fibrosis, CF; cystic fibrosis, CTD-ILD; connective tissue disease associated interstitial lung disease, IPAH; idiopathic pulmonary artery hypertension.

**TABLE 3:** Performance of 1-year mortality prediction model after lung transplantation with 25 features.

Model	AUC	Accuracy	Sensitivity	Specificity	PPV	NPV
LR	0.884 ± 0.005	0.881 ± 0.006	0.792 ± 0.006	0.898 ± 0.007	0.600 ± 0.017	0.958 ± 0.001
SVM	0.870 ± 0.004	0.917 ± 0.002	0.790 ± 0.006	0.941 ± 0.003	0.719 ± 0.009	0.959 ± 0.001
RF	0.951 ± 0.004	0.948 ± 0.002	0.731 ± 0.011	0.989 ± 0.002	0.929 ± 0.012	0.950 ± 0.002
GBM	0.958 ± 0.002	0.949 ± 0.002	0.756 ± 0.003	0.986 ± 0.002	0.911 ± 0.011	0.955 ± 0.001
BRF	0.954 ± 0.003	0.939 ± 0.002	0.788 ± 0.006	0.968 ± 0.002	0.826 ± 0.010	0.960 ± 0.001
MLP	0.924 ± 0.005	0.930 ± 0.004	0.649 ± 0.020	0.984 ± 0.002	0.886 ± 0.015	0.936 ± 0.003

This Table summarizes the performance indicators of various prediction models using 25 factors for 1-year mortality after lung transplantation.

AUC: area under the curve, PPV: positive predictive value, NPV: negative predictive value, LR: logistic regression, RF: random forest, SVM: support vector machine, GBM: gradient boosting machine, MLP: multilayer perceptron, BRF: balanced random forest.

Feature importance was analyzed using SHAP, with Figure 2 displaying the feature importance for the GBM model. The most important feature was albumin, with lower levels associated with higher mortality 1-year post-transplant. Other significant predictors included mean pulmonary artery pressure, creatinine levels, total bilirubin, and age.

Based on the SHAP results, we trained a new model using the top-10 most important features (Table 4). Despite reducing the number of features from 25 to 10, the performances of the six models remained largely unchanged. Some models, including LR, RF, SVM, and MLP, exhibited slight improvements in AUC, although accuracy was slightly decreased. The GBM model continued to demonstrate the highest performance with an AUC and accuracy of 0.958 and 0.949, respectively, even with only 10 features.

**TABLE 4:** Performance of 1-year mortality prediction model after lung transplantation with 10 features.

Model	AUC	Accuracy	Sensitivity	Specificity	PPV	NPV
LR	0.882 ± 0.005	0.884 ± 0.005	0.786 ± 0.003	0.902 ± 0.006	0.607 ± 0.015	0.956 ± 0.001
SVM	0.869 ± 0.005	0.917 ± 0.002	0.786 ± 0.006	0.942 ± 0.003	0.722 ± 0.010	0.958 ± 0.001
RF	0.951 ± 0.003	0.948 ± 0.002	0.735 ± 0.008	0.989 ± 0.002	0.926 ± 0.013	0.951 ± 0.001
GBM	0.958 ± 0.002	0.949 ± 0.002	0.755 ± 0.003	0.986 ± 0.002	0.914 ± 0.009	0.955 ± 0.001
BRF	0.953 ± 0.002	0.938 ± 0.002	0.791 ± 0.005	0.967 ± 0.002	0.821 ± 0.009	0.960 ± 0.001
MLP	0.939 ± 0.005	0.935 ± 0.001	0.664 ± 0.008	0.987 ± 0.002	0.906 ± 0.011	0.939 ± 0.001

This Table summarizes the performance indicators of various prediction models using 10 factors for 1-year mortality after lung transplantation.

AUC: area under the curve, PPV: positive predictive value, NPV: negative predictive value, LR: logistic regression, RF: random forest, SVM: support vector machine, GBM: gradient boosting machine, MLP: multilayer perceptron, BRF: balanced random forest.

### Actual Survival Curves: Low-Risk vs. High-Risk Groups by the GBM Model

**FIGURE 3:**
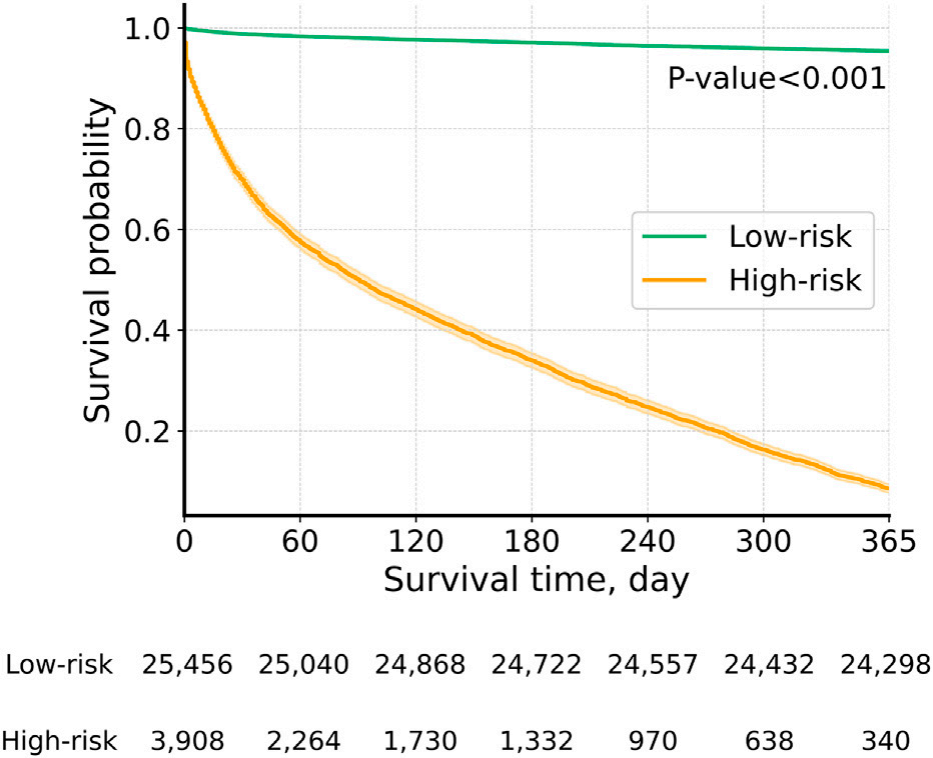
Actual survival curves: low-risk vs. high-risk groups by the GBM model. Graph depicting actual survival curves of the group whose survival was predicted by the GBM model.

We further validated the GBM model by comparing the actual survival curves of patients who were classified into two groups based on the model’s predictions. Patients predicted by the model to die were assigned to the high-risk group (death group), while those predicted to survive were placed in the low-risk group (survival group). As shown in Figure 3, the survival curves demonstrated a statistically significant difference between the two groups (p < 0.001). Among the predicted high-risk group, 91.4% experienced actual mortality, while 95.5% of the predicted low-risk group survived. These findings highlight the model’s robust predictive performance for distinguishing between mortality and survival outcomes.

### External Validation on In-House Dataset of PNUYH

To evaluate the generalizability of our model, we conducted an external validation using data from Pusan National University Yangsan Hospital (PNUYH) (Table 5). The model was developed using all 29,364 samples included in this study and subsequently validated on the in-house dataset of PNUYH. Between January 2012 and March 2024, a total of 228 adult patients (aged ≥18 years) underwent lung transplantation at PNUYH. After excluding 12 patients who underwent retransplantation, 216 patients were included in the external validation cohort. Among them, 70 (32.4%) died within 1 year of transplantation. Key preoperative characteristics of these patients are summarized in Supplementary Table S1. Notably, the mortality group had a significantly higher BMI compared to the survival group (23.2 vs. 21.3, p = 0.001). Serum albumin levels were lower in the mortality group (2.5 vs. 3.7, p < 0.001), and steroid use was more frequent (47.1% vs. 24.0%, p = 0.001). Additionally, a history of lung surgery prior to transplantation was more common in the mortality group (5.7% vs. 0.7%, p = 0.021).

**TABLE 5:** Results of External Validation for 1-Year Mortality Prediction Model After Lung Transplantation Using 10 Features on in-house dataset of PNUYH.

Model	AUC	Accuracy	Sensitivity	Specificity	PPV	NPV
LR	0.905	0.708	0.986	0.575	0.527	0.988
SVM	0.936	0.750	0.957	0.651	0.568	0.969
RF	0.870	0.769	0.443	0.925	0.738	0.776
GBM	0.852	0.764	0.529	0.877	0.673	0.795
BRF	0.888	0.815	0.857	0.795	0.667	0.921
MLP	0.911	0.870	0.886	0.863	0.756	0.940

This table summarizes the results of external validation on in-house dataset of PNUYH, using various prediction models with 10 features for 1-year mortality after lung transplantation.

AUC: area under the curve, PPV: positive predictive value, NPV: negative predictive value, LR: logistic regression, RF: random forest, SVM: support vector machine, GBM: gradient boosting machine, MLP: multilayer perceptron, BRF: balanced random forest.

The PNUYH dataset used for external validation had a significantly different distribution compared to the ISHLT dataset used for training (Supplementary Table S2), with all 10 model variables showing statistically significant differences between the two cohorts. Despite these differences, the GBM, which had the highest performance in the ISHLT dataset, demonstrated excellent external validation results (AUC: 0.852, accuracy: 0.764). Among all tested models, the highest AUC was observed for the SVM model (0.936). However, when considering both AUC and accuracy, the best-performing model was the MLP, a deep learning-based approach. The MLP achieved an AUC of 0.911 and an accuracy of 0.870, consistently outperforming other models across all evaluation metrics. Given the substantial distributional differences between the ISHLT and PNUYH datasets, the MLP model’s strong generalization performance underscores its robustness. These findings highlight the model’s ability to maintain high predictive performance in an external population, supporting its potential clinical utility in lung transplant candidate selection.

## Discussion

In this study, we developed and validated a deep learning-based model to predict 1-year survival following lung transplantation using a large, multicenter, international dataset. Our model demonstrated strong predictive performance, effectively identifying key determinants of post-transplant survival. By leveraging GBM techniques, we constructed a highly accurate and robust prediction model. Notably, a simplified version of our model, incorporating only the 10 most influential predictors, achieved performance comparable to that of more complex models utilizing 25 variables. Furthermore, the model’s generalizability was confirmed through external validation using the in-house dataset from PNUYH. The GBM model achieved an AUC of 0.852 and an accuracy of 0.764, and the MLP model demonstrated superior performance, achieving an AUC of 0.911 and accuracy of 0.870. These findings underscore the potential clinical applicability of our model in improving risk stratification and decision-making for lung transplant recipients.

The 10 predictors identified in this study were critical in assessing the potential benefit of lung transplantation. Among these, age, BMI, creatinine levels, total bilirubin levels, and mean pulmonary artery pressure have been well-established as critical indicators for assessing patient urgency and potential benefit in previous Lung Allocation Scores (LAS) [22] and the current Composite Allocation Score (CAS) [23]. Factors such as albumin levels, chronic steroid use, and prior lung surgery, newly highlighted in our study, further emphasize their potential to refine patient assessments and improve prediction accuracy. Albumin is traditionally a marker of nutritional status and inflammation, with lower levels associated with poorer post-transplant outcomes in previous studies [24]. This underscores the importance of evaluating nutritional and inflammatory status during pretransplant assessments. Chronic steroid use, identified in earlier studies, increases post-transplant morbidity and mortality [25], with some suggesting that long-term steroid use may be a contraindication for surgery [26]. Long-term steroid usage increases the risk of infection, poor wound healing, and other complications, all of which may be important predictors of transplant outcomes. Furthermore, prior major lung resection has been recognized as a significant risk factor for increased perioperative mortality and complications such as the need for dialysis [27]. This increased risk can be attributed to factors such as altered anatomy, potential for adhesions, and bleeding, all of which complicate the transplant procedure. These findings highlight the importance of thorough preoperative assessment in patients with a history of major lung resection.

Traditionally, prognosis following lung transplantation has been significantly influenced by the underlying primary disease necessitating the transplant. Different lung diseases impact post-transplant outcomes due to their distinct pathophysiology, patient demographics, and associated comorbidities. For example, patients with cystic fibrosis (CF) generally exhibit better post-transplant survival rates compared to those with idiopathic pulmonary fibrosis or chronic obstructive pulmonary disease [8]. In that regard, patient diagnosis plays an important role in both LAS and CAS, and several studies have incorporated diagnoses into prediction models. In our study, only cystic fibrosis was included as a predictor, but it had the lowest importance in the model (Figure 2B). Additionally, no patients with cystic fibrosis were included in the PNUYH in-house dataset used for external validation. Nevertheless, our model demonstrated excellent predictive performance, suggesting that preoperative conditions, such as organ function, nutritional status, and preoperative hospitalization, may be more critical prognostic indicators than the underlying disease itself. Thus, general aspects of patient health prior to transplantation could be more important than the specific underlying disease in pretransplant management.

While several studies have attempted to predict mortality in lung transplant patients, accurate predictions have not always been achieved due to limited performance (Supplementary Table S3) [28–30]. A recent study accurately predicted 1-year survival using 22 factors, including postoperative variables such as operation time, donor PaO2/FiO2 ratio, postoperative ECMO time, ventilator time, ICU stay, primary graft dysfunction grade, and cold ischemic time, achieving an AUC of 0.921 in patients from a single center [9]. Although these factors provide valuable insights into post-transplant outcomes, they are not available preoperatively, limiting their utility in pretransplant decision-making and patient prioritization. Our study addresses this limitation by focusing on preoperative variables that can be assessed before transplantation, thereby enhancing the ability to predict transplant outcomes and prioritize patients more effectively. Notably, achieving similar predictive accuracy with fewer variables has significant implications for clinical practice, as it simplifies the assessment process and makes it more feasible to implement in diverse healthcare settings without compromising predictive power. Furthermore, the external validation using in-house datasets further underscores the high generalizability of our model. Despite the fundamental differences between the training and validation datasets, the model demonstrated excellent performance, emphasizing its robustness and applicability across varied populations.

The use of machine learning approaches, particularly GBM, in this study highlights the transformative potential of these methods in healthcare. Machine learning models can handle complex interactions between variables and provide more accurate predictions compared to traditional statistical methods. This study demonstrates how machine learning models, such as GBM, can capture nonlinear relationships between variables, such as pulmonary artery pressure and BMI, which may not follow linear patterns. By incorporating these nonlinear interactions, the predictive performance of the model is significantly improved compared to traditional methods. SHAP values were used to visually explore the interactions between variables, providing insights into which characteristics contribute most to predictions. This ability to visualize complex interactions enhances the interpretability of the model, offering a deeper understanding of its decision-making process. Furthermore, comparing linear models (e.g., logistic regression, support vector machines) to nonlinear models (e.g., random forest, XGBoost) illustrates how traditional methods may miss out on capturing nonlinear patterns, which are crucial for accurate prediction of post-transplant outcomes. Our study advocates for the integration of machine learning technologies into clinical workflows. This integration can enhance clinical decision-making, providing more accurate predictions and improving patient outcomes. By leveraging machine learning models, clinicians can identify high-risk patients and tailor pretransplant management strategies to optimize post-transplant survival.

One significant limitation of our study was its reliance on registry data, meaning that the model’s performance depends on the accuracy and completeness of the recorded information. We mitigated this issue by excluding instances with missing data to use the most precise data available. We developed and internally validated the model using the ISHLT registry, which offers a large and diverse sample, and externally validated it using our own cohort, demonstrating the model’s generalizability. Future research should explore incorporating additional potential predictors and leveraging longitudinal data to further refine the model. These efforts would contribute to enhancing the model’s robustness and applicability in clinical practice. Additionally, further validation and ethical considerations should be conducted before applying the model to donor lung allocation, ensuring it addresses any ethical concerns.

In conclusion, our study confirmed that a machine learning-based approach can accurately predict 1-year mortality in lung transplant recipients using a minimal set of pretransplant factors. The development of a streamlined model with high predictive accuracy facilitates better patient selection, ensuring that lung transplantation resources are utilized efficiently and patient care is optimized. This model holds promise for enhancing clinical decision-making and improving post-transplant outcomes in lung transplant recipients. Investigating the underlying mechanisms by which specific pretransplant characteristics influence post-transplant outcomes could further enhance patient management strategies.

## Data Availability

The original contributions presented in the study are included in the article/Supplementary Material, further inquiries can be directed to the corresponding authors.

## Footnotes

1
https://ishlt.org/research-data/registries/ttx-registry

2
https://www.R-project.org/

## Abbreviations

AUC, area under the curve; GBM, Gradient Boosting Machine; ISHLT, International Society for Heart and Lung Transplantation; SHAP, Shapley Additive explanations.
