# Supplementary material for: Machine Learning for 1-Year Mortality Prediction in Lung Transplant Recipients: ISHLT Registry
Source: Transpl Int. 2025 Jun 26;38:14121. doi: 10.3389/ti.2025.14121 (PMC12234369; doi:10.3389/ti.2025.14121)
Supplement: Supplementary file 1 [file DataSheet1.docx]

Supplementary figure 1. Feature importance of 25 variables


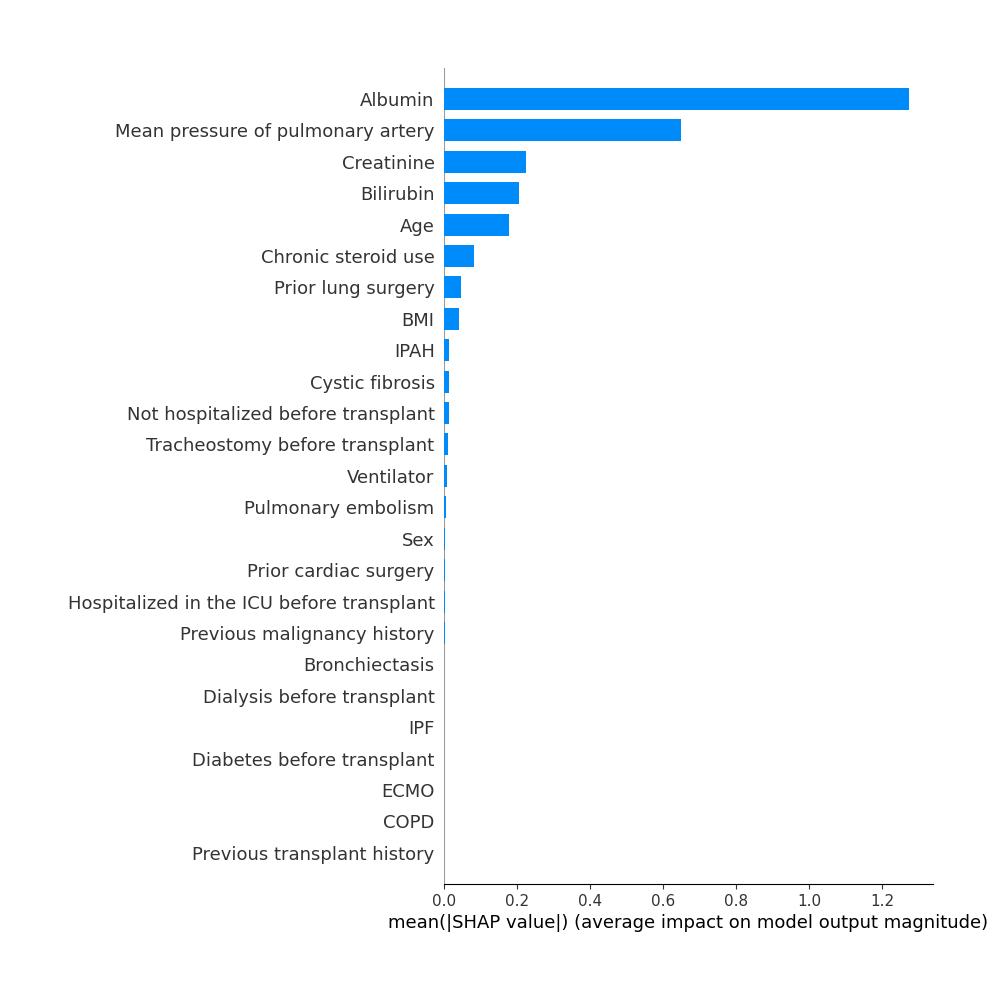


The size of the bar indicates the importance of each feature.

Supplementary figure 2. Five-Fold Cross-Validation Process


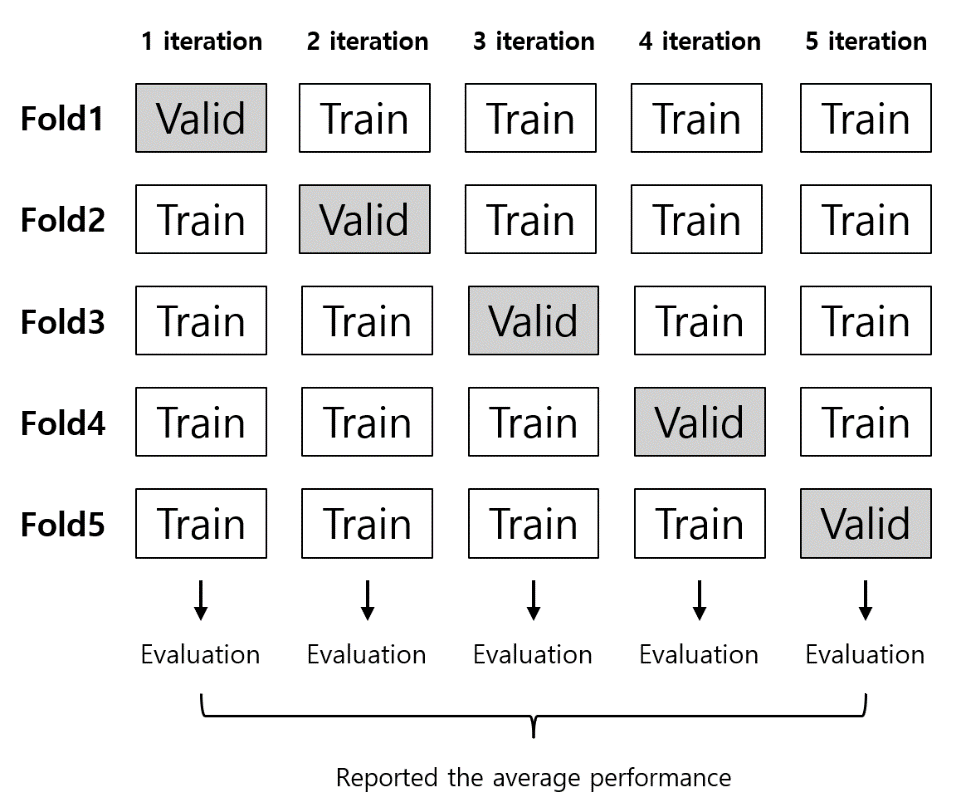


Five-fold cross-validation is a method used to evaluate the performance of a machine learning model. In this process, the entire dataset is divided into five equal subsets, or "folds." For each iteration of the cross-validation, one fold is reserved for validation, while the remaining four folds are used to train the model. This process is repeated five times, with each fold serving as the validation set exactly once. The final model performance is then reported as the average of the performance scores obtained from all five iterations, which helps provide a more robust and reliable estimate of the model's ability to generalize to unseen data.

Supplementary Table S1. Key preoperative characteristics of the In-House Dataset of PNUYH

| Variable | Survivors (N=146) | Death (N=70) | P |
| --- | --- | --- | --- |
| Age | 56.5±9.7 | 57.7±8.8 | 0.385 |
| BMI, kg/m^2^ | 21.3±3.9 | 23.2±3.8 | 0.001 |
| Male | 104 (71.2) | 50 (71.4) | 0.976 |
| Diagnosis |  |  | 0.520 |
| Idiopathic pulmonary fibrosis | 78 (53.4) | 30 (42.9) |  |
| Connective tissue disease related interstitial lung disease | 8 (5.5) | 4 (5.7) |  |
| Other interstitial lung disease | 24 (16.4) | 20 (28.6) |  |
| Post-acute respiratory distress syndrome fibrosis | 12 (8.2) | 7 (10.0) |  |
| Bronchiolitis obliterans syndrome after hematopoietic cell transplantation | 9 (6.2) | 4 (5.7) |  |
| Chronic obstructive pulmonary disease | 8 (5.5) | 2 (2.9) |  |
| Primary pulmonary hypertension | 2 (1.4) | 0 |  |
| Lymphangioleiomyomatosis | 1 (0.7) | 0 |  |
| Bronchiectasis | 4 (2.7) | 3 (4.3) |  |
| Albumin, g/dL | 3.7±0.5 | 2.5±0.4 | <0.001 |
| Creatinine, mg/dL | 0.8±0.4 | 0.9±0.5 | 0.192 |
| Total bilirubin, mg/dL | 1.1 [0.7-1.7] | 1.4 [0.9-2.7] | 0.083 |
| Mean pulmonary artery pressure, mmHg | 36.1±15.6 | 40.7±15.2 | 0.074 |
| Chronic steroid use | 35 (24.0) | 33 (47.1) | 0.001 |
| Prior lung surgery | 1 (0.7) | 4 (5.7) | 0.021 |
| Medical condition before transplant |  |  | 0.391 |
| ICU admission | 86 (58.9) | 48 (68.6) |  |
| General ward admission | 19 (13.0) | 7 (10.0) |  |
| No admission | 41 (28.1) | 15 (21.4) |  |

Data presented as mean±SD, or median [IQR] or N(%). BMI; body mass index, ICU; intensive care unit.

Supplementary Table S2. Descriptive Statistics and Comparison of Key 10 Variables for ISHLT and PNUYH

| Variable | ISHLT (N=29364) | PNUYH (N=216) | P |
| --- | --- | --- | --- |
| Age | 53.40±13.43 | 56.85±9.40 | 0.004 |
| Albumin | 3.63±0.49 | 3.30±0.74 | <.001 |
| Bilirubin | 0.85±1.26 | 1.99±2.78 | <.001 |
| BMI | 24.93±4.51 | 21.89±3.97 | <.001 |
| Creatinine | 0.93±0.58 | 0.83±0.46 | 0.018 |
| Mean pressure of pulmonary artery | 28.65±8.66 | 35.68±14.32 | <.001 |
| Chronic steroid use | 7264 (24.7) | 68 (31.5) | 0.027 |
| Cystic fibrosis | 4290 (14.6) | 0 (0.0) | <.001 |
| Not hospitalized before transplant | 26092 (88.9) | 56 (25.9) | <.001 |
| Prior lung surgery | 2601 (8.9) | 5 (2.3) | 0.001 |
| Death | 4729 (16.1) | 70 (32.4) | <.001 |

Data presented as mean±SD or N(%). BMI; body mass index.

Supplementary Table S3. Comparison of prediction accuracy for survival after lung transplantation in each model

| Datasets | Model | **AUC** | **sensitivity** | **specificity** | **PPV** | **NPV** |
| --- | --- | --- | --- | --- | --- | --- |
| UNOS | Clinician | 0.61 | 0.52 | 0.67 | 0.22 | 0.89 |
|  | LASSO | 0.61 | 0.41 | 0.75 | 0.22 | 0.90 |
|  | Random Forest | 0.62 | 0.44 | 0.76 | 0.24 | 0.89 |
|  | Chan et al | 0.59 | 0.48 | 0.68 | 0.21 | 0.88 |
|  | LAS | 0.55 | 0.44 | 0.66 | 0.18 | 0.87 |
| **ISHLT** | **Random Forest (Ours)** | 0.95 | 0.74 | **0.99** | **0.92** | **0.95** |
|  | **GBM (Ours)** | **0.96** | **0.76** | **0.99** | 0.91 | **0.95** |

The lung allocation score and other available models lack predictive accuracy for post-lung transplant survival.

AUC: Area Under the Curve, PPV: Positive Predictive Value, NPV: Negative Predictive Value, UNOS: United Network for Organ Sharing, LASSO: Least Absolute Shrinkage and Selection Operator, LAS: Lung Allocation Score, ISHLT: International Society for Heart and Lung Transplantation, GBM: Gradient Boosting Machine
